# Associations between quality of life, physical activity, worry, depression and insomnia: A cross-sectional designed study in healthy pregnant women

**DOI:** 10.1371/journal.pone.0178181

**Published:** 2017-05-22

**Authors:** Danielle Mourady, Sami Richa, Rita Karam, Tatiana Papazian, Fabienne Hajj Moussa, Nada El Osta, Assaad Kesrouani, Joseph Azouri, Hicham Jabbour, Aline Hajj, Lydia Rabbaa Khabbaz

**Affiliations:** 1 Laboratoire de pharmacologie, pharmacie clinique et contrôle de qualité des médicaments, Faculty of Pharmacy, Saint-Joseph University of Beirut, Beirut, Lebanon; 2 Faculty of Pharmacy, Saint-Joseph University of Beirut, Beirut, Lebanon; 3 Faculty of Medicine, Saint-Joseph University of Beirut, Beirut, Lebanon; 4 Department of Psychiatry, Hôtel-Dieu de France Hospital, Saint-Joseph University of Beirut, Beirut, Lebanon; 5 Laboratory of Experimental and Clinical Pharmacology (LECP), Faculty of Sciences, Lebanese University, Beirut, Lebanon; 6 Quality assurance of pharmaceutical products program, Ministry of Public Health, Beirut, Lebanon; 7 Department of Public Health, Faculty of Medicine, Saint-Joseph University, Beirut, Lebanon; 8 Department of Obstetrics and Gynecology, Hôtel-Dieu de France Hospital, Saint-Joseph University of Beirut, Beirut, Lebanon; 9 Department of Obstetrics and Gynecology, Mont-Liban Hospital, Beirut, Lebanon; 10 Department of Anesthesia and Critical Care, Hôtel-Dieu de France Hospital, Saint-Joseph University of Beirut, Beirut, Lebanon; Associazione OASI Maria SS, ITALY

## Abstract

Health-related quality of life (QOL) is reported to be reduced during pregnancy. Associations between QOL, physical activity (PA), insomnia, depression and worry are insufficiently investigated among pregnant women. The aim of this study was to evaluate QOL and PA patterns among healthy pregnant women, and to examine how QOL might correlate to PA, sleep, worry and depression. This is an observational cross-sectional study, conducted among a convenient sample of 141 healthy pregnant women using five questionnaires: WHOQOL-brief (WHO quality of life questionnaire, brief version, ISI (Insomnia Severity Index), PSWQ (Penn State Worry Questionnaire), ZSRDS (Zung Self-Rating Depression Scale), and Pregnancy Physical Activity Questionnaire (PPAQ). Pre-gestational BMI was inversely correlated to overall health while education was positively correlated to psychological health, social relationships and environment domains. Smoking before and during pregnancy significantly impacted the general health and psychological health. Total and light PA were positively correlated to psychological health and social relationships. Sports/exercise showed positive correlations with several QOL domains. Insomnia and depression were significantly associated with a decrease in all domains of QOL, while worries were associated with a decrease in physical, psychological and environmental domains. There were significant negative correlations between ZSRDS scores and total activity. PA, worries, depression and insomnia affected QOL during pregnancy. Furthermore, pregnant women presenting depression had a reduced total PA. Sleep and mental health as well as encouraging PA during pregnancy are necessary to improve the quality of life of pregnant women.

## Introduction

Several factors can affect the quality of life of pregnant women, in particular sleep alteration, worries, anxiety, depression and excessive weight gain, which can be due to reduced physical activity [1]. Studies examining quality of life in healthy pregnant women are lacking. Most of the conducted research was done among women suffering from diseases such as hypertension or diabetes [2].

Regular physical activity (PA) during pregnancy improves quality of life and promotes good physical and mental health [3]. The evolution of PA throughout the pregnancy is controversial: while some studies describe a trend toward a gradual decrease as the pregnancy progresses, other studies show a decrease in PA during the first quarter with a gradual increase and then stability until the end of pregnancy [4] or no significant differences in PA variability by trimester [5]. Based on what is published, there is a need to further investigate PA patterns during pregnancy and factors affecting it.

Insomnia during pregnancy is associated with a longer labor and an increased risk of C-section as well as cardiovascular diseases, diabetes, neurological disorders, respiratory problems and mood disorders [6] and can lead to a restriction in physical activities and a decreased quality of life [1].

Finally, relationships between PA in pregnancy and psychological well-being were examined in some studies [7] and findings suggested that in obese pregnant women, a depressed mood, but not pregnancy-related worries, may be associated with less physical activity. The authors concluded that the combined risk of poor mental health and low physical activity levels made women vulnerable to pregnancy complications.

In the Middle-East, publications related to QOL or PA during pregnancy are almost inexistent; studies examining insomnia, worry or anxiety, and depression in pregnant women are also scarce.

The present study has three aims: first, to fulfill the gap existing in the literature and evaluate QOL and PA patterns among healthy pregnant women. Second, to examine how QOL might correlate to PA, sleep, worry and depression.

Finally, to evaluate the impact of several socio-demographic parameters on QOL, PA and mental health problems during pregnancy.

## Materials and methods

### Study design and sample

This was an observational prospective cross-sectional study, conducted among a convenient sample of pregnant women during their routine visit to gynecologist clinics in Beyrouth (clinics were randomly selected to be representative of the capital city). Data collection was realized between July 2015 and April 2016.

One hundred and forty one Lebanese pregnant women were enrolled after giving their written informed consent. Sample size was set between 100 and 200 participants as described in similar studies [5,7]. Exclusion criteria were: age below 18 years, any known chronic medical condition and inability to read and understand the questionnaires.

### Ethical considerations

The study protocol was approved by the ethics committee of Saint-Joseph University in Beirut (reference number: USJ-2015-21).

### Data collection

The questionnaires were anonymous, confidential and intended to be self-administered. Socio-demographic, anthropometric and clinical data were also collected. The following questionnaires were used:

WHOQOL-brief **(**World Health Organization Quality Of Life scale- brief): it is the short version of the WHOQOL-100. It is a validated instrument to measure quality of life in patients. It was developed by the WHOQOL Group with fifteen international field centers, simultaneously, in an attempt to develop a quality of life assessment that would be applicable cross-culturally. This self-administered questionnaire contains two items related to the overall quality of life (question 1) and general health (question 2) and 24 items of satisfaction on the quality of life divided into four domains: physical health (7 items), mental health (6 items), social relationships (3 items) and environmental health (8 items). Each item is rated on a Likert scale of five points. The scores of each domain and the two items related to the overall quality of life and overall health are transformed linearly to scores on a 0–100 scale, 100 indicating the greatest quality of life [8,9].

ISI questionnaire (Insomnia Severity Index): it is a self-administered tool based on DSM IV diagnostic criteria for insomnia [10]. It measures over the past two weeks the perception of the severity of sleep problems and their daytime consequences. The questionnaire consists of seven questions that assess the severity of sleep maintenance and early awakening (waking-up at night or early morning), the current sleep satisfaction, impact on daily functioning disturbances appearance for others to deterioration of quality of life related to insomnia and the level of concern about current sleep difficulties. It can be self-administered. Each question is characterized by a response according to the Likert 5-point scale. A total is then calculated and the resulting value is between 0 and 28. The interpretation of scores is as follows: 0–7: lack of clinically significant insomnia; 8–14: subclinical insomnia; 15–21: moderate clinical insomnia; 22–28: severe clinical insomnia.

PSWQ Questionnaire (Penn State Worry Questionnaire) is a self-administered questionnaire validated by TJ Meyer et al. in 1990 [11]; it is designed to assess generalized pathological worries, considering the degree of excessiveness and uncontrollability. It was developed to evaluate an individual’s disposition to worry, as well as the frequency of the condition, its excess or intensity, as well as the tendency for the person to worry generally and not in one or a small number of situations. PSWQ is composed of 16 items rated on a Likert scale between 1 (not at all typical of me) to 5 points (very typical of me). Five items are negatively worded (items 1, 3, 8, 10 and 11). The sum of all the item scores gives a total that ranges from 16 to 80, where the higher the value, the higher the levels of pathological worry. A total resulting score higher than 44 represents a clinically significant level of worry (44–62: moderate level; ≥ 63: high level).

Self-administered ZSRDS (Zung Self-Rating Depression Scale) is an effective instrument for evaluating depressive disorders in patients and for monitoring clinical status following treatment. It includes 20 items built to the clinical criteria for diagnosis of depressive symptoms. The patient answers each question by checking the box that suits him most: a little time (which has a value of 1), part of the time (which has a value of 2), much of the time (which a value of 3) and most of the time (which has a value of 4). Ten questionnaire items are reversed (items 2, 5, 6, 11, 12, 14, 16, 17, 18 and 20). A total is then calculated by adding the points of each question and the value obtained is between 20 and 80. The interpretation of the obtained score is as follows: <50: absence of depression; 50–59: mild depression; 60–69: moderate depression; ≥70: severe depression [12,13].

The Pregnancy Physical activity questionnaire, PPAQ is a widely used, self-administered tool for the assessment and measurement of physical activity levels amongst pregnant women. The PPAQ is a semi-quantitative questionnaire that asks respondents to report usual physical activity during the past month and queries the time spent participating in 32 activities including household/care-giving (13 activities), occupational (5 activities), sports/exercise (8 activities), transportation (3 activities), and inactivity (3 activities). For every participant, the number of minutes spent in each reported activity type were multiplied by its MET intensity and summed to arrive at a measure of average weekly energy expenditure (MET-hrs/wk). MET intensity scores were based upon the Compendium of Physical Activities [14]. Average weekly energy expenditure was further classified into categories based on activity intensity and type. Intensity categories were defined as sedentary (<1.5 METs), light (1.5–2.9 METs), moderate (3.0–6.0 METs), and vigorous (>6.0 METs). Categories of activity type included household, occupational, sports and exercise, and transportation. A transcultural adaptation was performed in a pilot study on a sample of 45 healthy Lebanese pregnant women. Internal and external consistency of the questionnaires were assessed on our pre-study population. Intra-class correlation coefficient were superior to 0.7 indicating an excellent reproducibility for all questionnaires. Cronbach alpha were superior to 0.7 for all questionnaires indicating a good internal consistency.

### Statistical analysis

The statistical software SPSS for Windows version 18.0 (Chicago, IL, USA) was used for statistical analysis. The significance level chosen corresponds to a *p*-value ≤ 0.05. Socio-demographic characteristics and health status have been described for the entire sample. The mean and standard deviation were calculated to describe quantitative variables. Partial actual and percentage were calculated to describe the variables.

The Kolmogorov-Smirnov test was used to examine the normality of quantitative variables at the samples. One way analysis of variance followed by Bonferroni multiple comparisons were used for comparison of continuous variables among groups (more than 2 categories). If the normality of the distribution of the variables was not met, Kruskal-Wallis tests were used, followed by multiple comparison tests after adjustment of alpha error.

Student tests were used for comparison of quantitative variables between two groups. If the normality of the distribution of the variables was not met, Mann-Whitney tests were used. Fisher Exact tests and chi-square tests were used to compare qualitative variables between two or more groups.

## Results and discussion

### Participants’ characteristics

The characteristics of the participants are presented in Table 1. Among these participants, nine encountered medical problems during previous pregnancies such as premature contractions, and spontaneous abortions and ten participant encountered medical problems during previous deliveries such as premature delivery, hemorrhage, and still-birth.

**Table 1:** Participants’ characteristics, mean ± sd or number (percent).

Variables	Trimester 1 N = 38	Trimester 2 N = 53	Trimester 3 N = 50	Total N = 141
**Age (years)**	28.63±5.22	30.58±4.80	31.90±5.31	30.52±5.22
**Weight (Kg)**^a^	64.41±12.03	66.80±9.72	71.46±9.45	67.81±10.63
**Pre-gestational BMI (Kg/m**^**2**^**)**	23.31±3.86	22.59±3.14	22.66±2.87	22.81±3.25
**Weight gain (Kg)**^a^	2.86±1.78	5.89±3.24	10.73±4.87	6.79±4.81
**Weight gain / week (Kg / week)**	0.31±0.20	0.27±0.20	0.35±0.15	0.31±0.19
**Gestational week**^a^	9.45±2.83	20.25±4.48	30.94±4.25	21.13±9.36
**Number of previous deliveries**	0.58±0.79	0.70±0.80	0.90±0.95	0.74±0.86
**Pre-gestational BMI:**				
Underweight	0(0.0%)	2(3.8%)	1(2.0%)	3(2.1%)
Normal	29(76.3%)	40(75.5%)	39(78.0%)	108(76.6%)
Overweight	8(21.1%)	10(18.9%)	8(16.0%)	26(18.4%)
Obese	1(2.6%)	1(1.9%)	2(4.0%)	4(2.8%)
**Nulliparous**^a^	23(60.5%)	26(49.1%)	21(42.0%)	70(49.6%)
**Education:**				
Primary	6(15.8%)	8(15.1%)	4(8.0%)	18(12.8%)
Secondary	14(36.8%)	13(24.5%)	9(18.0%)	36(25.5%)
University	18(47.4%)	32(60.4%)	37(74.0%)	87(61.7%)
**Occupational category:**				
Workers	11(28.9%)	27(50.9%)	29(58.0%)	67(47.5%)
Housewives	27(71.1%)	26(49.1%)	21(42.0%)	74(52.5%)
**Smoking before pregnancy**	13(34.2%)	18(34.0%)	20(40.0%)	51(36.2%)
**Smoking during pregnancy**	0(0.0%)	1(1.9%)	4(8.0%)	5(3.5%)
**Caffeine intake**	14(36.8%)	27(50.9%)	30(60.0%)	71(50.4%)
**Alcohol intake**	5(13.2%)	6(11.3%)	2(4.0%)	13(9.2%)
**Medical problems encountered during previous pregnancies**	2(5.3%)	2(3.8%)	5(10.0%)	9(6.4%)
**Medical problems encountered during previous deliveries**	3(7.9%)	1(1.9%)	6(12.0%)	10(7.1%)
**Vitamins, Calcium and/or folic acid intake**	20(52.6%)	38(71.7%)	37(74.0%)	95(67.4%)

^a^Significant differences between the trimesters (*p*-value <0.0001; Kruskal-Wallis test followed by multiple comparison tests after adjustment of alpha error).

### Average questionnaires’ scores

Table 2 shows average scores (ISI, PSWQ, ZSDS and WHOQOL-brief) and Table 3 shows categorized results for insomnia, worry and depression (ISI, PSWQ, ZSDS). 30.5% of participants suffered from mild to moderate depression. No severe depression was self-reported. 64.5% presented moderate to high level of worry and only 25.5% of pregnant women presented lack of clinically significant insomnia.

**Table 2:** Average scores ± sd for insomnia, worry, depression and QOL domains (ISI, PSWQ, ZSDS, WHOQOL-Brief questionnaires) by trimester of pregnancy and total average scores ± sd.

Questionnaire	Trimester 1 N = 38	Trimester 2 N = 53	Trimester 3 N = 50	Total N = 141
**ISI**	10.61 ± 7.44	11.23 ± 6.10	13.24 ± 6.24	11.77 ± 6.58
**PSWQ**	46.95 ± 12.13	46.55 ± 9.58	48.36 ± 11.13	47.30 ± 10.82
**ZSDS**	46.08 ± 8.01	44.77 ± 8.23	45.44 ± 8.17	45.36 ± 8.11
**General quality of life**	65.79 ± 16.87	72.17 ± 16.01	73.00 ± 15.02	70.74 ± 16.08
**General health**	69.74 ± 14.42	72.64 ± 20.37	70.00 ± 20.83	70.92 ± 19.05
**Physical health**	62.22 ± 14.03	61.86 ± 14.94	56.50 ± 16.35	60.06 ± 15.35
**Psychological health**	60.75 ± 13.62	62.50 ± 15.31	64.75 ± 15.60	62.83 ± 14.96
**Social relationships**	66.01 ± 13.61	63.68 ± 15.51	64.00 ± 17.04	64.42 ± 15.52
**Environment**^a^	56.41 ± 15.48	58.90 ± 16.23	64.81 ± 15.02	60.33 ± 15.88

^a^*p*-value < 0.05; ANOVA test followed by Bonferroni multiple comparisons tests.

**Table 3:** Categorized results for insomnia (ISI), worry (PSWQ) and depression (ZSDS).

Questionnaire	Trimester 1 N = 38	Trimester 2 N = 53	Trimester 3 N = 50	Total N = 141
**ZSDS**				
Absence of depression	28(73.7%)	37(69.8%)	33(66.0%)	98(69.5%)
Mild depression	7(18.4%)	15(28.3%)	16(32.0%)	38(27.0%)
Moderate depression	3(7.9%)	1(1.9%)	1(2.0%)	5(3.5%)
**PSWQ**				
No clinically significant level of worry	14(36.8%)	21(39.6%)	15(30.0%)	50(35.5%)
Moderate level of worry	22(57.9%)	29(54.7%)	29(58.0%)	80(56.7%)
High level of worry	2(5.3%)	3(5.7%)	6(12.0%)	11(7.8%)
**ISI**				
Lack of clinically significant insomnia	13(34.2%)	14(26.4%)	9(18.0%)	36(25.5%)
Subclinical insomnia	13(34.2%)	25(47.2%)	19(38.0%)	57(40.4%)
Moderate clinical insomnia	8(21.1%)	11(20.8%)	17(34.0%)	36(25.5%)
Severe clinical insomnia	4(10.5%)	3(5.7%)	5(10.0%)	12(8.5%)

No significant differences between trimesters (*p*-values >0.05; Fisher exact test).

Physical activity scores per trimester along with total scores are summarized in S1 Table.

### Associations between participant’s characteristics and questionnaires scores

#### Factors impacting WHOQOL-brief

No differences between trimesters of pregnancy (*p*-values > 0.05) were seen, except for the environmental domain of the quality of life, which increased from trimester 1 to 3 (Table 2).

A significant association between pre-gestational BMI and general health was observed, the latter being lower in overweight women (*p*-value = 0.005), (Table 4).

**Table 4:** Significant associations between participant’s characteristics and quality of life domains (N = 141).

Variable	General QOL	General Health	Physical health	Psychological health	Social relationships	Environment
**Age**	NS	NS	NS	NS	NS	NS
**Pre-gestational BMI:**	NS	*p*-value = 0.005	NS	NS	NS	NS
Underweight (n = 3)		91.67±14.43				
Normal (n = 108)		72.69±17.27				
Overweight (n = 30)		62.50±22.51				
**Education:**	NS	NS	NS	*p*-value = 0.009	*p*-value = 0.019	p-value = 0.000
Primary (n = 18)				53.70±15.12	55.56±16.91	47.05±14.83
Secondary (n = 36)				61.57±13.66	63.43±13.84	56.77±15.26
University (n = 87)				65.23±14.80	66.67±15.36	64.55±14.56
**Smoking before/during:**	NS	*p*-value = 0.045	NS	*p*-value = 0.045	NS	NS
No/No		76.60±18.50		64.95±14.59		
Yes/No		65.22±19.35		58.33±15.34		
Yes/Yes		75.00±17.68		65.83±9.94		
**Weight gain/week**	NS	NS	NS	NS	NS	NS
**Occupational category:**	NS	NS	NS	NS	NS	NS
Worker						
Housewife						
**Number of previous deliveries:**	NS	NS	NS	*p*-value = 0.022	NS	*p*-value = 0.037
0				62.62±14.24		59.69±15.33
1				64.15±16.14		63.01±16.32
2				65.22±11.96		60.73±16.66
3				43.33±16.82		44.38±6.77
**Medical problems encountered during previous deliveries:**	NS	NS	NS	*p*-value = 0.046	NS	NS
Yes (n = 10)				71.67±13.00		
No (n = 131)				62.15±14.93		

Kruskal-Wallis followed by multiple comparison tests after adjustment of alpha error, Mann-Whitney tests and Spearman correlations (NS = non-significant associations, *p*-value>0.05).

Significant positive associations between education level on one hand and psychological (*p*-value = 0.009), social (*p*-value = 0.019), or environmental (*p*-value = 0.000) domains of QOL on the other hand were observed, (Table 4).

Smoking significantly affected general health (*p*-value = 0.045) and the psychological (*p*-value = 0.045) domain of QOL with lower score among women who used to smoke before getting pregnant and stopped smoking during pregnancy (Table 4).

Number of previous deliveries significantly affected the psychological (*p*-value = 0.022) and environmental (*p*-value = 0.037) domains of QOL, with lower scores seen among women with three compared to none, one and two previous deliveries (Table 4).

#### Factors impacting PPAC scores

For total activity as well as activity by intensity or by type, no statistically significant differences were seen between trimesters of pregnancy (*p*-values > 0.05, Kruskal-Wallis tests), (S1 Table). However, statistically significant differences in PA were found between nulliparous and multiparous participants (Table 5).

**Table 5:** Summary of activity scores for nulliparous and multiparous participants (PPAC).

Activity	Nulliparous N = 70	Multiparous N = 71	*p*-value^a^
**Total activity**	124.32±74.01	178.75±86.87	**0.000**
**By intensity**			
Sedentary (<1.5 METs)	13.99±11.37	8.59±8.85	**0.002**
Light (1.5 to <3.0 METs)	85.72±49.08	120.11±58.64	**0.000**
Moderate (3.0–6.0 METS)	23.70±36.75	49.45±39.41	**0.000**
Vigorous (>6.0 METs)	0.91±3.67	0.59±2.07	0.531
**By type**			
Household/care giving	51.20±49.81	109.38±59.74	**0.000**
Occupational^b^	69.43±49.84	60.40±30.66	0.366
Sports/exercise	4.15±9.10	3.94±6.73	0.875
Transportation	17.02±14.29	18.12±18.69	0.695
Inactivity	22.19±17.18	15.84±16.45	**0.027**

^**a**^Values in bold are significant, Mann-Whitney and Student t tests

^b^Among working women (n = 67).

Age was significantly and positively correlated to total physical activity in pregnant women (correlation 0.179, *p*-value = 0.034) as well as to light, and moderate intensity activity, and to household/care giving activity type; it was negatively associated with sedentary activity and inactivity (S2 Table).

The number of previous deliveries significantly impacted PPAC scores: it was positively associated with total, light, moderate intensity and household/care giving type, while a negative association was found with sedentary activity and inactivity (S2 Table).

No significant associations were seen between PPAC scores and pre-gestational BMI, gestational week, insomnia, or worry.

Educational level, occupational category, caffeine and alcohol intake as well as encountering medical problems during previous deliveries, significantly influenced PPAC scores: total activity was higher in women with university degree and in those working. Sports/exercise scores were lower in caffeine consumers and transportation type activity scores lower in alcohol consumers (S3 Table).

Smoking (cigarette / hookah) had no effect on PPAC nor had vitamins, calcium or folic acid intake.

#### Factors impacting ZSRDS, PSWQ and ISI scores

There was a significant correlation between the level of education and ISI scores (*p*-value = 0.028), (Table 6).

**Table 6:** Relationship between education level of pregnant women and insomnia, worry, or depression (N = 141).

Questionnaire	Education	N	Mean (sd)	*p*-value
**ISI**	Primary	18	15.56±5.93	**0.028**
	Secondary	36	10.75±6.40	
	University	87	11.41±6.58	
**PSWQ**	Primary	18	49.06±9.56	0.365
	Secondary	36	45.19±9.47	
	University	87	47.80±11.54	
**ZSDS**	Primary	18	52.33±7.55	**0.000**
	Secondary	36	45.50±6.48	
	University	87	43.86±8.13	

Numbers in bold represent significant values (ANOVA and Kruskal-Wallis tests followed by multiple comparison tests after adjustment of alpha error).

A significant relationship was also observed between ZSRDS scores and the level of education (*p*-value = 0.000), (Table 6). ZSRDS scores were also significantly associated with professional status (*p*-value = 0.038); working women presented a lower average score (43.88 ± 7.59) compared to non-working women (46.70 ± 8.37).

Smoking (cigarette / hookah) before or during the pregnancy were both significantly associated to depression (*p*-value = 0.005) and women who never smoked had the lowest ZSDS scores (49.40 ± 4.34), (S4 Table).

There was a significant positive relationship between caffeine consumption during pregnancy and PSWQ (*p*-value = 0.048).

Finally, being a nulliparous or taking vitamin supplements, calcium and / or folic acid during pregnancy did not affect any score.

#### Correlations between the different questionnaires

Insomnia and depression were significantly associated with a decrease in all domains of QOL, while worries where associated with a decrease in physical, psychological and environmental domains (Table 7).

**Table 7:** Associations between insomnia, worry, depression and quality of life in pregnant women (N = 141).

Questionnaire	Spearman correlation coefficients	PSWQ	ISI	ZSDS
**ISI**	Correlation	0.298	1	
	*p*-value	**0.000**		
**ZSDS**	Correlation	0.326	0.552	1
	*p*-value	**0.000**	**0.000**	
**WHOQOL-brief:**				
General QOL	Correlation	-0.136	-0.264	-0.399
	*p*-value	0.107	**0.002**	**0.000**
General Health	Correlation	0.012	-0.274	-0.335
	*p*-value	0.888	**0.001**	**0.000**
Physical health	Correlation	-0.261	-0.634	-0.653
	*p*-value	**0.002**	**0.000**	**0.000**
Psychological Health	Correlation	-0.250	-0.444	-0.613
	*p*-value	**0.003**	**0.000**	**0.000**
Social relationships	Correlation	-0.126	-0.342	-0.427
	*p*-value	0.137	**0.000**	**0.000**
Environment	Correlation	-0.175	-0.342	-0.427
	*p*-value	**0.038**	**0.000**	**0.000**

Numbers in bold represent significant *p*-values.

Furthermore, insomnia, worries and depression were significantly associated (Table 7).

Several QOL domains were associated with PA (Table 8): total and light intensity PA were positively significantly associated to the psychological domain of quality of life and social relationships; while sedentary PA was significantly associated with social relationships only. Sports /exercise was the main type of PA that showed a significant association with the majority of quality of life domains such as general quality of life, physical and psychological health, social relationships and environment.

**Table 8:** Association between total activity, activity by intensity, activity by type and quality of life domains (N = 141 except for occupational activity, where N = 67).

		Activity	by	Intensity				Activity	by	Type	
WHOQOL-brief domains		Total activity	Sedentary	Light	Moderate	Vigorous	Household/care giving	Occupational	Sports/ exercise	Transportation	Inactivity
General QOL	Correl	0.082	0.046	0.065	0.063	0.110	0.069	0.091	0.194	0.015	-0.055
	*p*-value	0.331	0.585	0.445	0.459	0.193	0.418	0.464	**0.021**	0.860	0.518
General Health	Correl	0.089	0.044	0.035	0.121	0.098	0.047	0.022	0.146	0.110	0.003
	*p*-value	0.293	0.604	0.683	0.152	0.248	0.578	0.859	0.084	0.192	0.976
Physical health	Correl	0.005	-0.003	-0.043	0.066	0.080	0.041	-0.100	0.202	0.009	-0.084
	*p*-value	0.950	0.973	0.616	0.435	0.343	0.632	0.421	**0.016**	0.912	0.320
Psychological health	Correl	0.194	0.068	0.201	0.104	0.061	0.089	0.160	0.210	0.051	0.026
	*p*-value	**0.021**	0.426	**0.017**	0.219	0.472	0.293	0.195	**0.012**	0.550	0.758
Social relationships	Correl	0.219	0.194	0.203	0.144	0.059	0.102	0.267	0.206	0.044	0.064
	*p*-value	**0.009**	**0.021**	**0.016**	0.087	0.488	0.227	0.029	**0.014**	0.602	0.449
Environment	Correl	0.125	0.042	0.092	0.121	0.022	0.018	**0.175**	0.202	0.132	-0.010
	*p*-value	0.141	0.621	0.279	0.151	0.793	0.833	0.156	**0.016**	0.118	0.903

Spearman correlation coefficients (Correl) are represented along with *p*-values. Values in bold represent significant results (*p-*value<0.05).

There were significant negative correlations between ZSRDS scores and total activity, as well as with light intensity activity and sports/exercise activity type (S2 Table). However, no associations were found between PA and worry nor between PA and insomnia.

Our study is one of the few evaluating QOL as well as PA during the three trimesters of pregnancy as well as factors affecting it, and the first to explore their association with three mental health correlates: insomnia, worry and depression.

Previous studies reported health-related quality of life to be reduced during pregnancy in all trimesters [15] with a decrease in the second and third, compared to the first trimester [16].

Our results did not show differences in reported QOL among trimesters, except for the environment domain with scores increasing from trimester 1 to 3. This could reflect an improvement in the environment’s safety and the access to health services as well as better transportation means with advanced pregnancy. Age and weight gain per week were not however, associated with any domain of the quality of life.

Pre-gestational BMI was inversely correlated to overall health in our study. Encouraging obese women to have a normal weight before planning to conceive can help them to better perceive their general health and improve their quality of life.

Education but not working status was associated in previous findings with health-related quality of life during pregnancy [16]. Our results were consistent with these findings since education was positively correlated to psychological health, social relationships and environment domains, while occupational category was not associated with any domain of the WHOQOL-brief.

The results of our study also reveals that smoking (cigarette / hookah) before and during pregnancy significantly impacts the general health and psychological domain of the QOL. Women who used to smoke and stopped when pregnant showed the lowest reported scores.

Women with three children and more presented the lowest psychological health and environment score. Encountering a medical problem during previous deliveries was associated with a higher score in the psychological domain of QOL which could be explained by the fact that 7 out of the 10 women with previous medical problems were being followed up by a psychologist during current pregnancy.

Psychiatric disorders can affect the psychological as well as the physical domains of the QOL during pregnancy [16]. This was also observed in our study since increased worry was associated with a decrease in two domains of QOL: environment and psychological while depression was associated with a decrease in all QOL domains. Furthermore, pregnant women with high ISI scores (insomnia) showed a decrease in all quality of life domains.

PA in pregnancy is well known to increase well-being, self-esteem and improve body’s image [17].

Previous findings on PA evolution across pregnancy trimesters are conflicting: moderate to vigorous activity was reported to drop between gestational week 15 and 28 [18] while other research reported a decrease of self-reported PA from the first to the second and from the first to third trimester (total, light and moderate intensity activities were concerned) while time spent on most activities remained fairly stable throughout pregnancy [19]. Our findings revealed that there were no significant differences in total PA of pregnant women among trimesters. Neither PA by intensity nor by type showed any statistical difference among trimesters of pregnancy.

Age was positively correlated to some components of PA (total, light and moderate): when age increases, light and moderate intensity activity increase while sedentary activity and inactivity decrease. Time spent on household/care giving activities increases with women’s age. This interesting finding could be related to misconceptions about PA especially among younger pregnant women. Better knowledge of the benefits of PA during pregnancy is necessary to avoid false beliefs that pregnant women should rest, leading to being sedentary and inactive.

Weight gain per week showed a significant negative association with vigorous PA as well as with sports/exercise type of activity. This is an important finding since it shows that more importantly than actual weight of the pregnant woman or pre-gestational BMI, weight gained each week could be a predictive factor leading to reduced exercising, thus leading to a secondary increase in weight and creating a vicious circle hard to break.

Having children at home was positively associated to PA in previous studies [20]. In our study, number of previous deliveries was positively associated with total activity, light, and moderate intensity PA as well as with household/care giving type. Furthermore, women with no offspring presented lower total activity, as well as lower light and moderate intensity activities than multiparous women. As for activity type: household/care giving were more important in multiparous, which was expected since they are supposed to care for children at home, in contrary to nulliparous women.

Existing literature on the association between education and PA in pregnancy is inconclusive: higher education among western women is reported to be positively associated to PA, while among South Asian women, higher education and PA are inversely related [20]. Our results showed that education is positively associated with PA, and total PA increases with the women education level.

Workers have higher PA, which was somehow expected. Caffeine and alcohol seem to impact activity type but not intensity: caffeine intake lowers sports/exercise type of PA while alcohol lowers transportation type. No clear explanation can be provided at this level of our research knowing that such findings were not described in previous studies.

Many studies investigated the benefits of PA on the promotion of health and QOL in different diseases such as kidney diseases, osteoporosis, cancer and cardiovascular diseases [21] but very few were conducted among pregnant women and results are contradictory [21,22].

In this study, total PA as well as light PA was positively correlated to psychological health and social relationships domain of QOL, while sedentary PA was positively correlated to social relationships only (sedentary pregnant women could have more time on hand to spend socializing). When analyzing activity by type, sports/exercise showed positive correlations with 5 out of 6 domains of QOL. These combined results clearly highlight the need to strongly encourage light sports/exercise activity among women aiming to improve their QOL during pregnancy.

As for insomnia during pregnancy, its prevalence was reported to be around 57% with no significant variation throughout the period of pregnancy [23]. Our results showed that 40.4% of pregnant women suffer from subclinical insomnia, 25.5% from moderate clinical insomnia and 8.5% suffer from severe clinical insomnia with no significant differences between pregnancy trimesters, nor between nulliparous and multiparous women.

Insomnia was associated with a lowest education level and smoking (smoking during pregnancy was associated with higher ISI scores). On the other hand, the higher the education level of pregnant women, the lower insomnia and depression scores. According to a study in the USA, the prevalence of clinical insomnia is higher when the level of education is lower [24] as confirmed in our findings.

Insomnia was also significantly associated with worry, depression and all domains of the quality of life but no significant associations were observed with PA.

With reference to anxiety, this study specifically focused on detecting maternal worries, which are the cognitive components of anxiety and the key symptoms of generalized anxiety disorder (GAD).

A study conducted in Spain [25] showed that the intensity of worries decreases from the first to the third trimester but other studies revealed that worry drops in mid-pregnancy and rises again at 35 weeks [26].

Our results showed a prevalence of 56.7% (for moderate level of worry) and 7.8% (high level of worry) in pregnant women with no statistically significant differences among trimesters. Worry was associated with insomnia and depression, as well as with caffeine consumption, but not with nulliparity nor to PA.

One predictor of low physical activity levels that has been well studied in the general population is poor emotional well-being signifying depression.

Our results showed that 30.5% of pregnant women suffer from depression, mainly mild. This is comparable to prevalence rates in the Unites states (33%), Turkey (27.9%) [27] and Brazil (up to 28%) but higher than in Asia (17.5%) or Europe (19.5%) [28].

Research indicates that performing physical activity during pregnancy can reduce instances of depression [29]. In our study, there was a significant association between PA and depression: women who scored higher on the ZSDS questionnaire showed lower total physical activity and were less prone to sports and exercise.

According to a previous study [30], well-educated pregnant women have a lower risk of emotional disorders (especially depression, anxiety and melancholy) and physical disorders (pain and discomfort) related to compensation through work, a better economic status, personal control of their life. Our findings revealed that depression was associated with worry and insomnia as well as with lower education level; pregnant women with higher education presented less depression.

Another result observed in our study is that less depression is seen in working women as compared to women who do not work. Other studies do confirm that risk factors for gestational depression are mostly linked to indicators of socioeconomic deprivation, such as unemployment [28].

Smoking is known to be a risk factor for perinatal depression and is associated with psychosocial difficulties [31]. Depression rates were reported to be 12.9% for women who never smoked, 25.1% for women who smoked before pregnancy and 37.5% for women who still smoked during pregnancy. In our study, women smoking cigarette / hookah before and during pregnancy had a higher prevalence of depression when compared to women who never smoked.

Regarding relations between the three mental health markers examined, i.e. insomnia, worry and depression, our results clearly showed that the three are intimately associated: pregnant women presenting one of these psychological problems are likely to present the other two. Mental health screening during pregnancy should take this finding into consideration.

Our results do not contradict the literature where associations were previously found between insomnia, depression and anxiety in pregnant women [32].

## Conclusions

There are several limitations to this study that should be taken into consideration. First, the cross-sectional design limited interpretations about causality. Second, and although all questionnaires are validated and were completed anonymously, they represent self-reported perceptions about insomnia, mental health, PA and QOL, and not objective measures. In addition, our study only considers Lebanon’s capital; the use of a convenient sample due to practical limitations should also be noted. Finally, the quality of sleep needs to be explored and not only insomnia, in future studies.

Despite these limitations, this research contributes to the general body of knowledge on QOL and impacting factors during pregnancy and it is to best of our knowledge, the first to evaluate relations between QOL, three mental health components and PA in pregnant women.

The results presented should contribute to improving the quality of life of pregnant women by taking into consideration factors impacting it, as well as acknowledging the significant associations between sleep and mental health during pregnancy.

## Supporting information

S1 Table Summary of activity scores per trimester and total scores (PPAC). No significant differences between trimesters (*p*-values > 0.05, Kruskal-Wallis test).
(DOCX)

S2 Table Significant associations between participant’s characteristics (quantitative variables), ZSDS scores, total activity, activity by intensity and activity by type (N = 141 except for occupational activity, where N = 67). Spearman correlation coefficients are presented (NS = non-significant associations, *p*-value>0.05).
(DOCX)

S3 Table Significant associations between participant’s characteristics (qualitative variables), total activity, activity by intensity and activity by type (N = 141). Kruskal-Wallis followed by multiple comparison tests after adjustment of alpha error and Mann-Whitney tests
(NS = non-significant associations, *p*-value>0.05).
(DOCX)

S4 Table Associations of smoking status before and during pregnancy with insomnia, worry, and depression (N = 141). Numbers in bold represent significant values (ANOVA followed by Bonferroni multiple comparison tests and Kruskal-Wallis tests followed by multiple comparison tests after adjustment of alpha error).
(DOCX)
