# Supplementary material for: Associations between quality of life, physical activity, worry, depression and insomnia: A cross-sectional designed study in healthy pregnant women
Source: PLoS One. 2017 May 22;12(5):e0178181. doi: 10.1371/journal.pone.0178181 (PMC5439948; doi:10.1371/journal.pone.0178181)
Supplement: S1 Table — No significant differences between trimesters (p-values > 0.05, Kruskal-Wallis test). (DOCX) [file pone.0178181.s001.docx]

S 1Table. Summary of activity scores per trimester and total scores (PPAC).

| Activity | Trimester 1  N=38 | Trimester 2  N=53 | Trimester  N=50 | Total  N=141 |
| --- | --- | --- | --- | --- |
| Total activity | 132.09 ± 77.92 | 145.43 ± 76.25 | 173.33 ± 95.15 | 151.73 ± 84.97 |
| **By intensity**  Sedentary (< 1.5 METs)  Light (1.5-<3.0 METs)  Moderate activity (3.0-6.0 METs)  Vigorous activity (>6.0 METs) | 11.29 ± 7.94  89.88 ± 53.50  30.47 ± 34.85  0.46 ± 1.41 | 11.30 ± 11.79  99.51 ± 52.42  34.03 ± 34.08  0.59 ± 1.96 | 11.23 ± 10.94  116.79 ± 61.13  44.18 ± 48.50  1.14 ± 4.39 | 11.27 ± 10.50  103.04 ± 56.61  36.67 ± 40.11  0.75 ± 2.97 |
| **By type**  Household/care giving activity  Occupational activity^a^  Sports/exercice activity  Transportation activity  Inactivity | 75.60 ± 53.77  67.74 ± 32.84  3.09 ± 4.22  14.26 ± 14.94  17.74 ± 11.78 | 75.06 ± 57.79  59.47 ± 35.62  3.21 ± 7.58  17.35 ± 14.91  19.52 ± 19.11 | 89.97 ± 71.81  67.83 ± 47.63  5.65 ± 10.14  20.34 ± 19.14  19.38 ± 18.37 | 80.49 ± 62.12  64.44 ± 40.31  4.04 ± 7.97  17.57 ± 16.60  18.99 ± 17.06 |

No significant differences between trimesters (*p*-values > 0.05, Kruskal-Wallis test)

^a^Among working women (n=67)
