# Supplementary material for: Associations between quality of life, physical activity, worry, depression and insomnia: A cross-sectional designed study in healthy pregnant women
Source: PLoS One. 2017 May 22;12(5):e0178181. doi: 10.1371/journal.pone.0178181 (PMC5439948; doi:10.1371/journal.pone.0178181)
Supplement: S2 Table — Spearman correlation coefficients are presented (NS = non-significant associations, p-value>0.05). (DOCX) [file pone.0178181.s002.docx]

S2 Table. Significant associations between participant’s characteristics (quantitative variables), ZSDS scores, total activity, activity by intensity and

activity by type (N=141 except for occupational activity, where N=67).

| **Variable** |  | **Activity** | **by** | **intensity** |  |  | **Activity** | **by** | **type** |  |
| --- | --- | --- | --- | --- | --- | --- | --- | --- | --- | --- |
|  | Total activity | Sedentary | Light | Moderate | Vigorous | Household/  care giving | Occupational | Sports/  exercice | Transportation | Inactivity |
| **Age (years)** | Correlation = .179  *p*-value=.034 | Correlation = -.271  *p*-value=.001 | Correlation = .194  *p*-value=.021 | Correlation = .175  *p*-value=.038 | NS | Correlation = .200  *p*-value=.017 | NS | NS | NS | Correlation = -.178  *p*-value=.034 |
| **Weight gain/week** | NS | NS | NS | NS | Correlation = -.187  *p*-value=.027 | NS | NS | Correlation = -.307  *p*-value=.000 | NS | NS |
| **Number of previous deliveries** | Correlation = .220  *p*-value=.009 | Correlation =-.268  *p*-value=.001 | Correlation = .234  *p*-value=.005 | Correlation = .261  *p*-value=.002 | NS | Correlation = .424  *p*-value=.000 | NS | NS | NS | Correlation = -.190  *p*-value=.024 |
| **ZSDS** | Correlation = -.176  *p*-value=.037 | NS | Correlation = -.182  *p-*value=.031 | NS | NS | NS | NS | Correlation = -.193  *p*-value=.022 | NS | NS |

Spearman correlation coefficients are presented (NS=non-significant associations, *p*-value>0.05).
