# Supplementary material for: Associations between quality of life, physical activity, worry, depression and insomnia: A cross-sectional designed study in healthy pregnant women
Source: PLoS One. 2017 May 22;12(5):e0178181. doi: 10.1371/journal.pone.0178181 (PMC5439948; doi:10.1371/journal.pone.0178181)
Supplement: S3 Table — Kruskal-Wallis followed by multiple comparison tests after adjustment of alpha error and Mann-Whitney tests (NS = non-significant associations, p-value>0.05). (DOCX) [file pone.0178181.s003.docx]

S3 Table. Significant associations between participant’s characteristics (qualitative variables), totalactivity, activity by intensity and activity by type (N=141).

| **Variable** | **Total activity** | **Activity by intensity** | **Activity by** | **type** |
| --- | --- | --- | --- | --- |
|  |  | Vigorous | Sports/exercise | Transportation |
| **Education:**  Primary  Secondary  University | *p*-value= 0.006  97.70±49.00^a^  143.77±80.12^b^  166.19±88.41^b^ | NS | NS | NS |
| **Occupational category:**  Workers  Housewives | *p*-value=0.000  185.97±86.45  120.72±70.97 | NS | NS | NS |
| **Caffeine intake:**  Yes  No | NS | NS | *p*-value=0.014  2.83±4.62  5.27±10.20 | NS |
| **Alcohol intake:**  Yes  No | NS | NS | NS | *p*-value=0.003  10.44±6.83  18.30±17.14 |
| **Medical problems encountered during previous pregnancies:**  Yes  No | NS | *p*-value=0.014  0.79±3.06  0.09±0.28 | NS | NS |

Kruskal-Wallis followed by multiple comparison tests after adjustment of alpha error and Mann-Whitney tests

(NS=non-significant associations, *p*-value>0.05).
