# Supplementary material for: Associations between quality of life, physical activity, worry, depression and insomnia: A cross-sectional designed study in healthy pregnant women
Source: PLoS One. 2017 May 22;12(5):e0178181. doi: 10.1371/journal.pone.0178181 (PMC5439948; doi:10.1371/journal.pone.0178181)
Supplement: S4 Table — Numbers in bold represent significant values (ANOVA followed by Bonferroni multiple comparison tests and Kruskal-Wallis tests followed by multiple comparison tests after adjustment of alpha error). (DOCX) [file pone.0178181.s004.docx]

S 4 Table. Associations of smoking status before and during pregnancy with insomnia,

worry, and depression (N=141).

| **Questionnaire** | **Smoking**  **before/during** | **N** | **Mean±sd** | ***p*-value** |
| --- | --- | --- | --- | --- |
| **ISI** | No/No  Yes/No  Yes/Yes | 90  46  5 | 10.86**±**6.27  13.33**±**7.15  14.00**±**3.74 | **0.086** |
| **PSWQ** | No/No  Yes/No  Yes/Yes | 90  46  5 | 46.42**±**11.41  48.61**±**9.42  51.00**±**12.15 | 0.399 |
| **ZSDS** | No/No  Yes/No  Yes/Yes | 90  46  5 | 43.71**±**8.15^a^  48.15**±**7.50^b^  49.40**±**4.34^b^ | **0.005** |

Numbers in bold represent significant values (ANOVA followed by Bonferroni multiple comparison

tests and Kruskal-Wallis tests followed by multiple comparison tests after adjustment of alpha error).
